# Characterization of Peptidyl-Prolyl *Cis-Trans* Isomerase- and Calmodulin-Binding Activity of a Cytosolic *Arabidopsis thaliana* Cyclophilin AtCyp19-3

**DOI:** 10.1371/journal.pone.0136692

**Published:** 2015-08-28

**Authors:** Gundeep Kaur, Supreet Singh, Harpreet Singh, Mrinalini Chawla, Tanima Dutta, Harsimran Kaur, Kyle Bender, W. A. Snedden, Sanjay Kapoor, Ashwani Pareek, Prabhjeet Singh

**Affiliations:** 1 Department of Biotechnology, Guru Nanak Dev University, Amritsar-143005, Punjab, India; 2 Department of Bioinformatics, Hans Raj Mahila Maha Vidayalaya, Jalandhar, Punjab, India; 3 Interdiscipinary Center for Plant Genomics and Department of Plant Molecular Biology, University of Delhi South Campus, New Delhi, India; 4 Department of Biology, Queen’s University, Kingston, Ontario, Canada; 5 Stress Physiology and Molecular Biology Laboratory, School of Life Sciences, Jawaharlal, Nehru University, New Delhi, India; Scripps Research Institute, UNITED STATES

## Abstract

Cyclophilins, which bind to immunosuppressant cyclosporin A (CsA), are ubiquitous proteins and constitute a multigene family in higher organisms. Several members of this family are reported to catalyze *cis-trans* isomerisation of the peptidyl-prolyl bond, which is a rate limiting step in protein folding. The physiological role of these proteins in plants, with few exceptions, is still a matter of speculation. Although *Arabidopsis* genome is predicted to contain 35 cyclophilin genes, biochemical characterization, imperative for understanding their cellular function(s), has been carried only for few of the members. The present study reports the biochemical characterization of an *Arabidopsis* cyclophilin, AtCyp19-3, which demonstrated that this protein is enzymatically active and possesses peptidyl-prolyl *cis-trans* isomerase (PPIase) activity that is specifically inhibited by CsA with an inhibition constant (K_i_) of 18.75 nM. The PPIase activity of AtCyp19-3 was also sensitive to Cu^2+^, which covalently reacts with the sulfhydryl groups, implying redox regulation. Further, using calmodulin (CaM) gel overlay assays it was demonstrated that *in vitro* interaction of AtCyp19-3 with CaM is Ca^2+^-dependent, and CaM-binding domain is localized to 35–70 amino acid residues in the N-terminus. Bimolecular fluorescence complementation assays showed that AtCyp19-3 interacts with CaM *in vivo* also, thus, validating the *in vitro* observations. However, the PPIase activity of the *Arabidopsis* cyclophilin was not affected by CaM. The implications of these findings are discussed in the context of Ca^2+^ signaling and cyclophilin activity in *Arabidopsis*.

## Introduction

A peptide bond in a folded protein can attain either a *trans* or *cis* conformation. The *trans* form is sterically favoured and therefore proteins are synthesized with peptide bonds in this form [1]. However, proline, due to its five-membered ring, has a unique ability to adopt either *cis* or *trans* states of the backbone torsion angle in the peptide backbone. Therefore, approximately 6.5% of peptide bonds preceding proline residues are present in the *cis* conformation [2]. For proper folding of proteins, the *cis* to *trans* isomerisation of peptide bonds is essential since *cis*-proline introduces bends within a protein, decreasing stability. The *cis*-*trans* population ratio at thermal equilibrium is dependent on the difference in free energy (ΔG) between *cis* and *trans* states. The relative large energy barrier (ΔG = 14–24 kcal/mol) makes the *cis-trans* isomerisation a slow and rate-limiting process. Peptidyl-prolyl *cis-trans* isomerases (PPIases) are the only enzymes known which stabilize this *cis-trans* transition, lower the activation energy of the stabilized product and accelerate the isomerisation process [3].

Majority of the PPIases have been categorized as immunophilins on the basis of their affinity for immunosuppressive ligands cyclosporin A (CsA) and FK506. Depending upon this affinity, the PPIases are designated as cyclophilins [4] or FK506-binding proteins (FKBPs) [5], which bind to CsA or FK506, respectively. The different immunophilins form complexes with these ligands and play an important role in mediating immunosuppression in animals [6]. A large number of cyclophilins have been identified in various organisms such as bacteria, yeast, fungi, mammals and plants [7]. Cyclophilins in plants were first discovered with the isolation of cyclophilin-encoding cDNA sequences from tomato (*Lycopersicon esculentum*), maize (*Zea mays*) and oilseed rape (*Brassica napus*) [8]. Compared to 28 in rice, the largest cyclophilin family identified in any organism to date has been reported in *Arabidopsis*, containing 35 cyclophilins, with predicted molecular weights and pIs ranging between 18–94 kDa and 4.5–12.4, respectively [9–12]. The members of cyclophilin family in *Arabidopsis* were first isolated and characterized by Chou and Gasser [7], and designated as ROC1 to ROC6 (rotamase cyclophilin). Romano et al. [10] identified several novel members of this gene family by *in silico* analysis, and also proposed a new nomenclature, wherein the cyclophilins were designated as AtCyp, suffixed with the molecular weight and the number of the gene encoding isoform.

The Arabidopsis genome is predicted to contain 13 different cytosolic cyclophilins out of which nine [AtCYP18-1, AtCYP18-2, AtCYP18-3 (ROC1), AtCYP18-4 (ROC5), AtCYP19-1 (ROC3), AtCYP19-2 (ROC6), AtCYP19-3 (ROC2), AtCYP22-1, AtCYP26-1)] consist of a single domain whereas four (AtCYP40, AtCYP57, AtCYP65, AtCYP71) show the presence of multiple domains [9, 10]. The single domain cyclophilins contain a single conserved cyclophilin-like domain that may or may not contain the N-terminal targeting sequence. The multidomain members of this family also contain additional domains such as the U-box domain (AtCYP65) [10], tetratricopeptide repeats (AtCyp40) [13], WD repeats (AtCyp71) [10], the Arg/Ser (RS)-rich domain, the S/K-R/E-rich domain and the Glu-Lys (EK) domain (AtCYP57) [10, 14]. Though AtCyp19-3 is closely related to other single domain cyclophilins such as AtCYP18-3, AtCYP18-4, AtCYP19-1, AtCYP19-2 and hCypA, two unique amino acid substitutions in AtCYP19-3 (G108K, E128N) account for its divergence from these proteins [10]. Though the physiological role of cyclophilins in plants is still a matter of speculation [15], recent studies have implicated some of these proteins in a diverse range of functions such as protein folding [16], chloroplast biogenesis and photosynthesis [17], plant growth and development [18, 19], redox regulation [20] and stress response [21–24]. In *Arabidopsis*, the cytosolic AtCyp18-3 [25] and AtCyp19-1 [26] were found to function in plant-pathogen interaction, with the former also participating in brassinosteroid and gibberellic acid signalling [27]. The chloroplast-localized isoform, AtCyp20-3 (ROC4), was demonstrated to play a vital role in cysteine biosynthesis [20]. A survey of literature (Table 1) indicates that characterization of biochemical activity, which is imperative for understanding the physiological significance of cyclophilins in plants, has been carried out only for few members of this family [16, 23, 28–35]. Of the 35 different cyclophilin genes identified in *Arabidopsis* [9, 10, 12], biochemical characterization has been reported only for AtCyp20-2 [36], AtCyp22 [28] and AtCyp38 [37]. One of the cytosolic cyclophilins, AtCyp19-3 (ROC2), was demonstrated to interact with CaM [38], which is a transducer of intracellular changes in [Ca^2+^] [39], thereby, suggesting that this protein may be playing an important role in signal transduction in the cell. However, information as to whether AtCyp19-3 is capable of catalysing *cis-trans* isomerization is lacking. The wheat homologue, TaCypA-1, which shares about 70% identity with AtCyp19-3, was recently demonstrated to possess PPIase activity [29]. Recent studies have shown that despite high levels of sequence identity, the biochemical properties of cyclophilins, including PPIase activity, are not conserved in plants [37], indicating divergence in their physiological roles during evolution. Thus in the present study, we characterized the PPIase activity of AtCyp19-3 so as to better understand the role of this protein in the plants. Furthermore, the CaM-binding properties of AtCyp19-3 were explored and are discussed in the context of a possible role for AtCyp19-3 in Ca^2+^-mediated signal transduction.

**Table 1:** Comparative analysis of biochemical parameters of various cyclophilins.

Organism	Generic name	Catalytic efficiency K_cat_/K_m_ (M^-1^ s^-1^)	Single domain (SD)/ Multidomain (MD)	Inhibition constant (k_i_) for CsA (nM)	References	
*Arabidopsis thaliana*	*AtCyp19-3*	2.7x10^6^	SD	18.75	This study	
*Ricinus communis*	*RcCyp1*	9.48x10^6^	SD	-	Gottschalk *et al*., 2008	[16]
*Oryza sativa*	*OsCyp2*	4.5 x10^6^	SD	-	Kumari *et al*., 2009	[23]
*Arabidopsis thaliana*	*AtCyp22*	5.7 x10^6^	SD	8.0	Grebe *et al*., 2000	[28]
*Triticum aestivum*	*TaCypA-1*	2.32x10^5^	SD	78.3	Sekhon *et al*., 2013	[29]
*Zea mays*	*ZmCyp18*	11x10^6^	SD	6.0	Sheldon and Venis, 1996	[30]
*Citrus sinensis*	*CsCyp*	5.6x10^6^	SD	-	Campos *et al*., 2013	[31]
*Spinacia oleracea*	*TLP40*	1.6x10^6^	MD	-	Fulgosi *et al*., 1998	[32]
*Homosapiens*	*Cyp40*	1.9x10^6^	MD	2–200	Kieffer *et al*., 1992	[33]
					Kofron *et al*., 1991	[34]
*Homosapiens*	*CypA*	1.4 x 10^7^	SD	2.9	Daum *et al*., 2009	[35]
*Homosapiens*	*CypB*	5.6 x 10^6^	SD	8.4	Daum *et al*., 2009	[35]

## Materials and Methods

The cDNAs encoding full length *AtCyp19-3* (At3g56070), procured from Arabidopsis Biological Resource Centre, and the truncated versions *AtCyp19-*3_(213–528)_ and *AtCyp19-3*_(105–528)_ encoding (AtCyp19-3_(71–176)_ and AtCyp19-3_(35–176)_, respectively) were subcloned into the expression vector pET-28a(+). The *E*.*coli* cyclophilin PpiA (NC_000913) that lacks cysteine [40, 41] residues was included in this study for comparative analysis. The gene encoding PpiA was amplified without stop codon by polymerase chain reaction (PCR) from genomic DNA that was isolated from TOP10 *E*.*coli* cells as described earlier [42]. Sequences of the different primers and the conditions used for PCR amplification of different gene fragments are listed in S1 Table. The PCR reaction mix contained primer (1 μM), dNTPs (1 mM) and Pfu polymerase (1 unit). The amplified products were analysed on agarose gels, extracted using a commercial gel extraction kit (Merck Specialities Pvt. Ltd., India) as per manufacturer’s instructions, and cloned into pET-28a(+) after digesting with *EcoR*I and *Xho*I (for *AtCyp19-3*) and *Bam*HI and *Xho*I (for *ppiA*). The sequences of the cloned fragments were confirmed by sequencing from Bioserve Biotechnologies (I) Pvt. Ltd., India.

### Induction and purification of the recombinant proteins

The full length and truncated recombinant AtCyp19-3 proteins were expressed as 6xHis fusion proteins in *E*. *coli* BL21(DE3)LysS cells after the induction of cultures with 0.5 mM isopropyl-β-D-1-thiogalactopyranoside (IPTG) when A_600_ reached 0.4–0.5, followed by further incubation at 25°C for 4 h with shaking at 200 rpm. The inducton of *E*.*coli* PpiA was also carried out similarly except that the cultures were grown at 37°C after addition of IPTG. The induced proteins were analyzed by SDS-PAGE [43] followed by staining with Coomassie Brilliant Blue-R250 (CBB). For purification of the recombinant proteins, the cells were lysed in lysis buffer (50 mM NaH_2_PO_4_-pH 8.0, 300 mM NaCl, 0.25% Triton X-100, 1 mM protease inhibitor cocktail (PIC), 10% glycerol, 10 mM imidazole) followed by sonication (time: 30 sec, pulse on/off: 1.0 sec). The lysed samples were centrifuged for 20 min at 14,000 g and the supernatants containing the recombinant fusion proteins were incubated with Ni-NTA slurry (G Biosciences, USA) in binding buffer (50 mM NaH_2_PO_4_-pH 8.0, 300 mM NaCl, 0.25% Triton X-100, 1 mM PIC, 10% glycerol, 10 mM imidazole) for 2 h at 4°C and applied on to the columns. The columns were washed with three bed-volumes of wash buffer (50 mM NaH_2_PO_4_-pH 8.0, 300 mM NaCl, 0.25% Triton X-100, 1 mM PIC, 10% glycerol, 50 mM imidazole) followed by centrifugation at 4000 g for 5 min. The matrix-bound proteins were eluted thrice by addition of one bed-volume of elution buffer (50 mM NaH_2_PO_4_-pH 8.0, 300 mM NaCl, 0.25% Triton X-100, 1 mM PIC, 10% glycerol, 250 mM imidazole). The purified recombinant proteins were analyzed by 12% SDS-PAGE and visualized following CBB staining. The purification of TaCypA-1 was performed as described earlier [29].

### Immunoblot analysis of recombinant proteins by anti-His antibodies

Immunoblotting of the purified recombinant proteins was carried out as described earlier [44]. The proteins were separated by 12% SDS-PAGE and transferred to Hybond C membrane (Amersham Pharmacia Biotech, England). The transfer buffer consisted of 150 mM glycine, 20 mM Tris-HCl (pH 8.0), 0.1% SDS, 10% methanol. After the transfer, the membrane was stained with Ponceau S (Sigma-Aldrich, USA). After destaining in autoclaved double distilled water, the membrane was incubated for 2 h at RT in blocking buffer (200 mM Tris-HCl-pH 7.5, 1.4 M NaCl, 0.02% Tween-20, 3% BSA). The blots were washed thrice with TBST (200 mM Tris-HCl-pH 7.5, 1.4 M NaCl, 0.02% Tween-20), followed by TBS (200 mM Tris-HCl-pH 7.5, 1.4 M NaCl) for 10 min with each of the buffers. The blots were incubated with rabbit anti-His antibodies (Biovision, India) at 1:10,000 dilution in blocking buffer for 2 h for AtCyp19-3 proteins and for overnight for PpiA. The blots were washed thrice with TBST containing 0.2% Tween-20, followed by TBS for 10 min each. After washing, the blots were incubated in alkaline-phosphatase conjugated goat anti-rabbit secondary antibodies (Sigma-Aldrich, USA) (diluted 1:10,000 in TBS buffer) for 2 h. After washing the blot thrice with TBST and TBS for 10 min each, the protein-antibody complex was visualized by incubating in 5-bromo-4-chloro-3-indolyl phosphate/nitroblue tetrazolium solution.

### Peptidyl prolyl *cis-trans* isomerase (PPIase) activity assay

The concentration of the purified recombinant proteins was estimated by Bradford method [45] using bovine serum albumin (BSA) as a standard. PPIase activity of the full length and truncated AtCyp19-3 proteins was assayed at 15°C for 360 s in a coupled reaction with chymotrypsin as described earlier [46]. The 1 ml assay mixture contained 80 μM succinyl-ala-ala-pro-phe-p-nitroanilidine as test peptide, assay buffer (50 mM HEPES-pH 8.0, 150 mM NaCl, 0.05% Triton X-100) and different concentrations of the purified proteins. The reaction was initiated by addition of chymotrypsin (300 μg/ml) and change in absorbance at 390 nm was monitored by using spectrophotomer (Perkin- Elmer Lambda Bio 25) equipped with peltier temperature control system. The effect of FK506 and CsA (specific inhibitors of PPIase activity of FKBPs and cyclophilins, respectively) was studied by determining the inhibition of the reaction. The CsA and FK506 were added to the assay mix containing purified cyclophilin (4.34 nM), followed by incubation at 4°C for 30 min before the start of reaction by addition of test peptide and chymotrypsin. The functional role of thiol groups in regulating the PPIase activity of purified AtCyp19-3, TaCypA-1 and PpiA was studied by incubating the proteins (22 nM) in assay buffer containing 500 μM N-ethylmaleimide (NEM) (diluted from 50 mg/ml stock prepared in 100% EtOH) for different time intervals and different concentrations of CuSO_4_ [47], followed by estimation of PPIase activity. The incubation of purified cyclophilins with CuSO_4_ was carried out on ice for 30 min. The PPIase activity was calculated as a product of the difference in catalysed and uncatalysed first order rate constant (derived from kinetics of absorbance change at 390 nm) and amount of substrate in each reaction [48]. The data obtained were analysed using Grafit 4.0 software (http://www.erithacus.com/grafit). The inhibition constants for CsA and Cu^2+^ were determined as gradient of the line of the best fit from a plot of (CsA)/(1-k/k_o_) against k_o_/k where k is the rate constant at any given CsA concentration and k_o_ is the rate constant in the absence of CsA [30].

### Calmodulin (CaM)-binding assays

CaM-binding property of the recombinant AtCyp19-3 proteins *in vitro* was studied by CaM gel-overlay assays. The CaM gel-overlay assays were carried out as described [49], except that the probing of the membrane with biotinylated CaM (Calbiochem, USA) for 1 h in binding buffer was carried out at 25°C instead of 4°C. For determining the role of CaM in regulation of enzyme activity of AtCyp19-3, the purified cyclophilin (22 nM) was incubated with CaM (100 nM and 200 nM) for 4 h at 25°C followed by estimation of PPIase activity as described earlier, except that the assay buffer contained 50 mM Tris (pH 7.5), 150 mM NaCl and 5 mM CaCl_2_. Bovine serum albumin (200 nM) was used as a control. For studying CaM-binding property *in vivo*, the coding regions of full-length *AtCyp19-3* and rice CaM (*OsCaM1*; LOC_Os03g20370), and the truncated *AtCyp19-3*_*(71–176)*_ genes, without stop codons, were cloned in pDH51-GW-YFP (N9841) vector for intracellular localization, and pDH51-GW-YFPn(N9842) and pDH51-GW-YFPc vectors (N9843) Gateway compatible vectors for bi-molecular fluorescence complementation (BiFC) analysis, respectively [50]. *OsCaM1* was PCR amplified using Phusion high-fidelity DNA polymerase (New England Biolabs Inc. USA), forward primer (5’CACCATGGCGGACCAGCTCACCGACGA3’) and reverse primer (5’CTTGGCCATCATGACCTTGACGAACTC3’) from cDNA prepared from rice seeds. A 447 bp amplicon thus obtained was gel extracted and cloned in pENTR/D-TOPO entry vector using pENTR directional TOPO cloning kit (Invitrogen Inc. USA). Similarly, *AtCyp19-3* and *AtCyp19-3*_*(71–176)*_ coding regions were PCR amplified from a pre-cloned *AtCyp19-3* cDNA in pET-28a(+) vector (forward primer, 5’caccATGGCGAATCCTAAAGTCTT3’; reverse primer 5’TGAACTTGGGTTCTTGAGCTC3’). The resultant amplicons of 528 and 318 bp, respectively, were gel-purified and subsequently cloned in pENTR/D-TOPO entry vector. For all the constructs, fidelity of inserts was validated by restriction digestion and nucleotide sequencing. The three protein coding regions were then mobilized into pDH51-GW-YFPn destination vector by using Gateway LR clonase kit (Invitrogen Inc. USA).

### Subcellular localization and BiFC assays

Onion epidermal cells were transfected by gene:reporter translational fusion constructs by particle bombardment using Biolistic PDS-1000/He particle delivery system (Bio-Rad, USA). Three milligrams of gold particles were washed thoroughly and coated with 2 μg of each plasmid in separate 1.5 ml microcentrifuge tubes [51]. The plasmid-coated micro carriers were bombarded on onion peel epidermal cells at 27 mm Hg vacuum, 1100 psi Hg pressure and 6 cm target distance. The plates were incubated in dark at 28°C for 16–18 h. The epi-fluorescence analysis to visualize YFP signals and data recording was carried out by using a LASER confocal microscope (TCS SP8, Leica Gmbh, Germany).

### Bioinformatics Analysis

Multiple sequence alignment of different cyclophilin proteins was performed using online clustalw2 server (https://www.ebi.ac.uk/Tools/msa/clustalw2/). The ESPript 3.0 multiple-alignment editor (http://espript.ibcp.fr/ESPript/ESPript/) was used for the final presentation of the multiple sequence alignment while the graphical view of the phylogenetic tree was obtained using Treeview. Comparative Modeling of AtCyp-19-3 was performed with modeller 9.12 using chain A of CsCYP (PDB ID 4JJM) as a template structure having 78% identity with the AtCyp-19-3 sequence. Five models were obtained and assessed using DOPE score and GA341 methods. Analysis of Metal Binding Site for AtCyp19-3 model, CsCYP (4JJM) and TaCypA-1(4E1Q) was performed using TEMSP Predict server (http://netalign.ustc.edu.cn/temsp/). Graphical images for various structural features and structure alignments were obtained with UCFC Chimera 1.81 [52] molecular visualization tool.

## Results and Discussion

**Fig 1:**
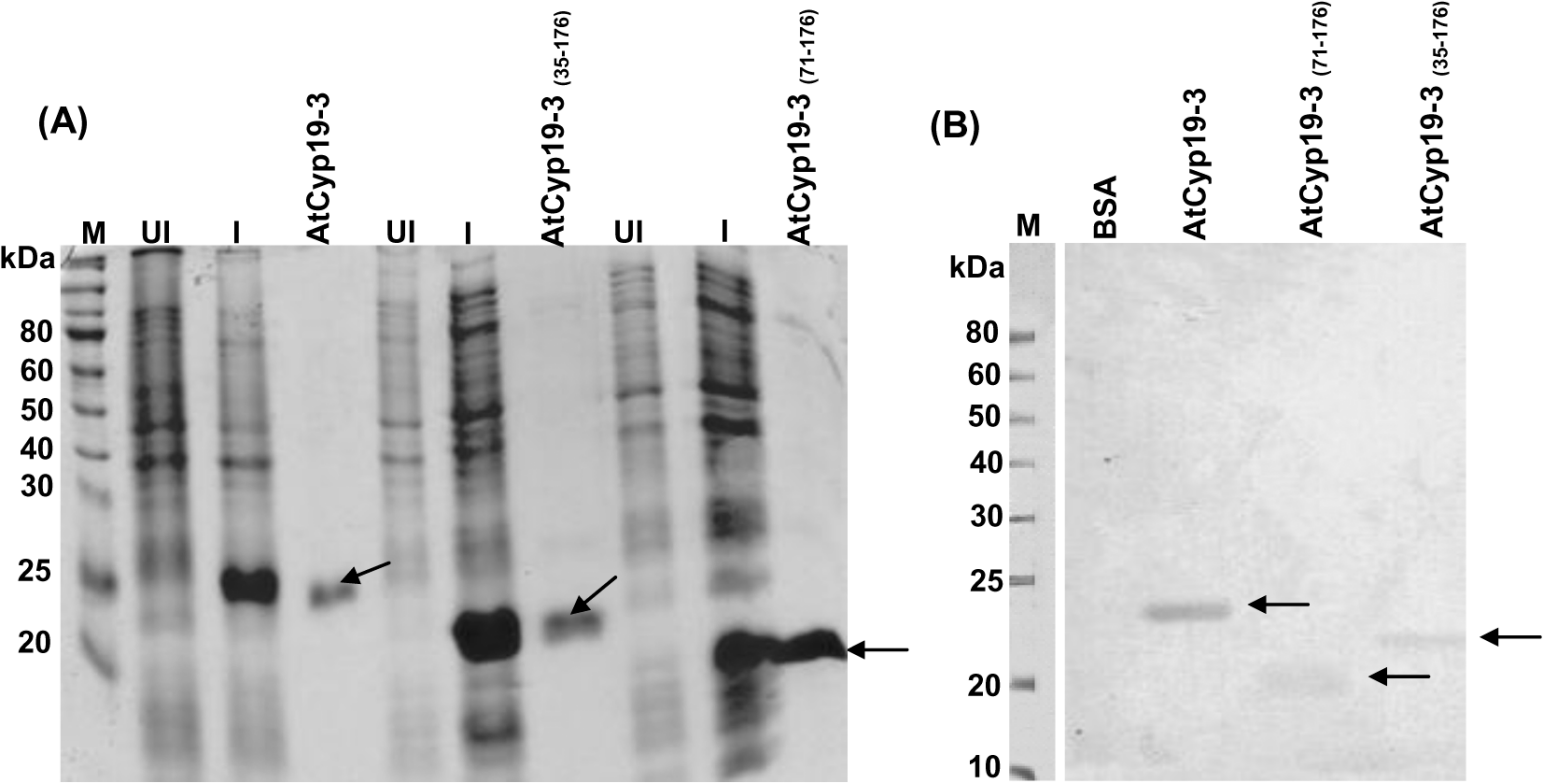
SDS-PAGE analysis of purified recombinant fusion proteins and their confirmation by immunoblotting with anti-His antibodies. **(A)** SDS-PAGE analysis of purified full length recombinant AtCyp19-3, and deleted versions AtCyp19-3 _(35–176)_ and AtCyp19-3 _(71–176)_. Total proteins were isolated from recombinant *E*.*coli* BL21(DE3) before (UI) and after induction (I) with 0.5 mM IPTG, and purified by Ni-NTA affinity column. Arrows indicate the purified recombinant proteins. **(B)** Confirmation of the purified recombinant proteins (arrows) by immunoblotting with the anti-His antibodies. M: markers.

As part of our ongoing studies to understand the functional significance of different cyclophilins, in the present study we examined the biochemical properties of AtCyp19-3 (ROC2), which shares 82.4% similarity with AtCyp18-3 (ROC1), 73.3% with AtCyp19-1(ROC3), 50.6% with AtCyp20-3 (ROC4), 81.8% with AtCyp18-4 (ROC5) and 86.4% with AtCyp19-2 (ROC6) (S2 Table) [7]. Except for AtCyp20-3, which is chloroplast-localized, the other cyclophilins have been reported to be cytosolic proteins [53]. For characterizing the different biochemical parameters, the purified full length AtCyp19-3 protein and its deletion mutants (AtCyp19-3_(35–176)_ and AtCyp19-3_(71–176)_) were obtained by cloning the full length (531 bp) and truncated cDNAs in the vector pET28a(+), followed by expression in *E*. *coli*. Recombinant full length AtCyp19-3, AtCyp19-3_(35–176)_ and AtCyp19-3_(71–176)_ were purified as ~23 kDa, 20 kDa and 15 kDa His-tagged fusion proteins, respectively (Fig 1A), consistent with the predicted MWs of 18.92 kDa, 15.15 kDa and 11.28 kDa, respectively. The recombinant nature of the full length and truncated proteins was confirmed by immunoblotting using anti-His antibodies which revealed the presence of specific bands for each of the three proteins (Fig 1B).

**Fig 2:**
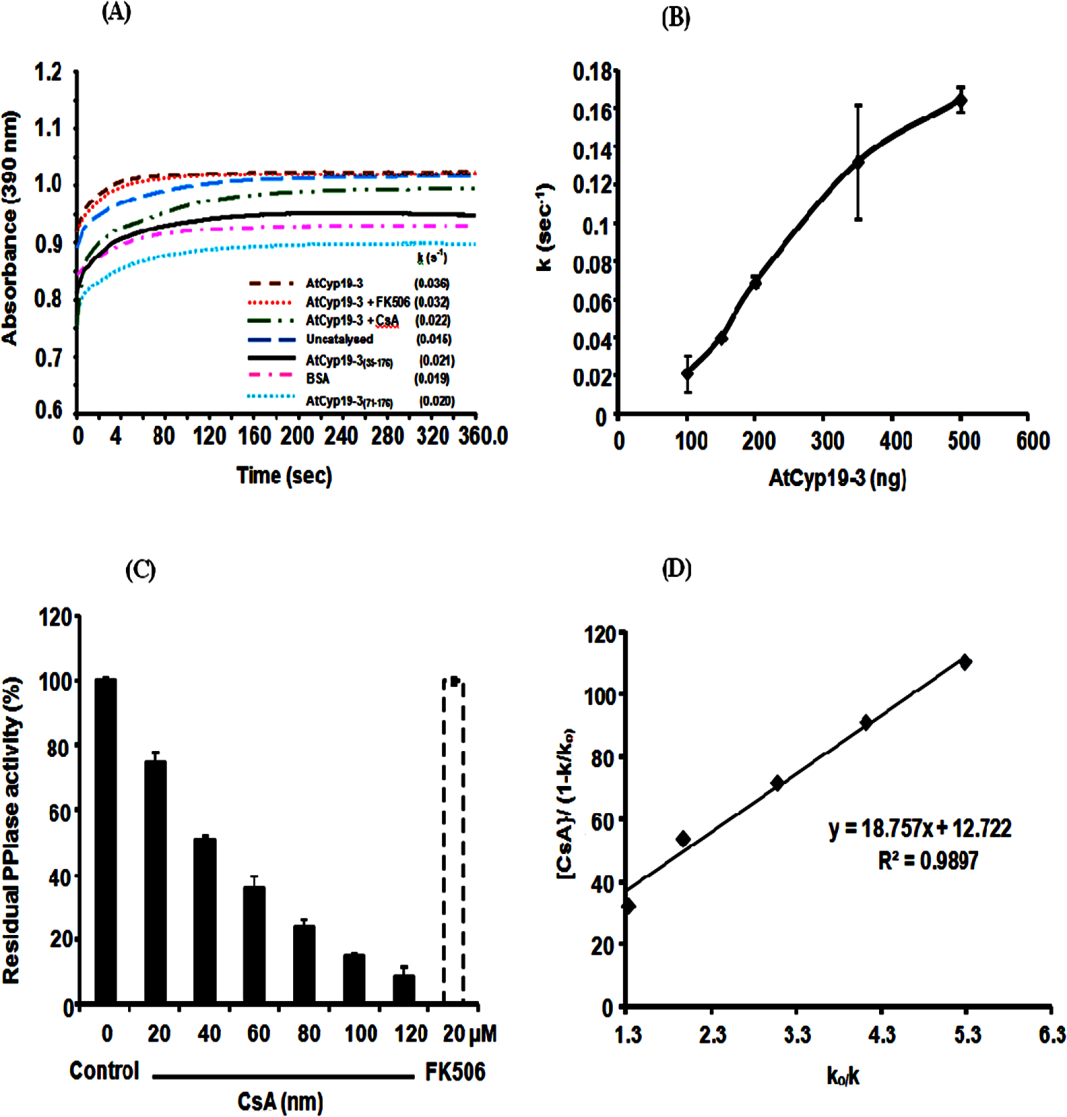
PPIase activity of purified recombinant fusion protein AtCyp19-3 **(A)** Hydrolysis of N-succinyl-ala-ala-pro-phe-p-nitroanilidine (peptidyl prolyl *cis-trans* isomerase or PPIase activity) in the presence of 4.34 nM of recombinant AtCyp19-3 and 52.4 nM and 65.87 nM each of AtCyp19-3_(35–176)_ and AtCyp19-3_(71–176)_ proteins. The rate of reaction is expressed as first order rate constant (k) Bovine serum albumin (BSA) was used as a negative control. **(B)** Effect of purified AtCyp19-3 on rate constant. **(C)** Effect of cyclophilin inhibitor, cyclosporin A (CsA), and FKBP inhibitor, FK506, on the PPIase activity of AtCyp19-3 **(D)** Determination of inhibition constant (k_i_) of AtCyp19-3 for CsA. Inhibition constant (k_i_) for CsA was determined as gradient of the line of the best fit from a plot of [CsA]/(1-k/k_o_) against k_o_/k, where k is the rate constant at any given CsA concentration and k_o_ is the rate constant in the absence of CsA. The slope of the line represents the k_i_.

**Fig 3:**
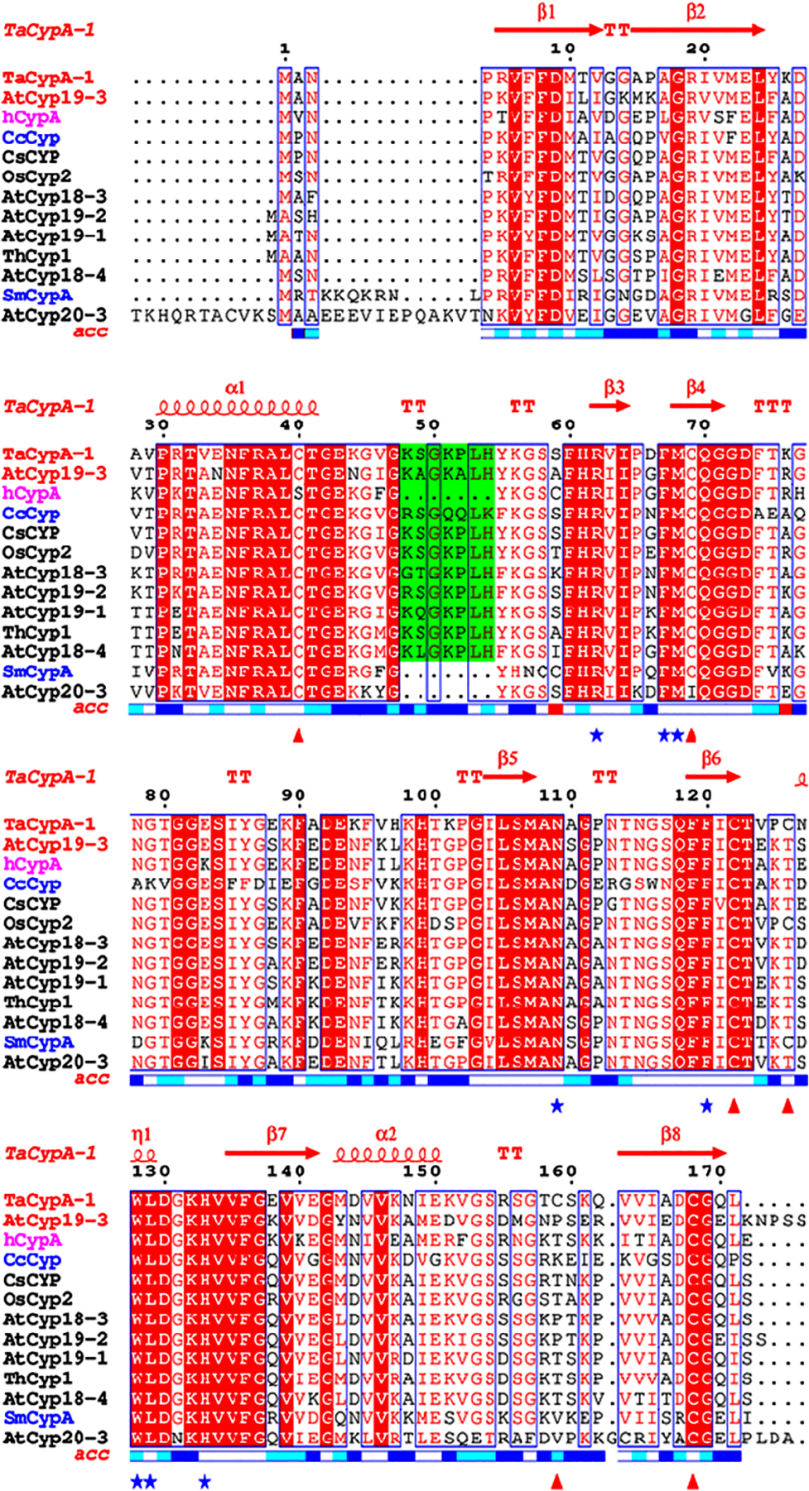
Multiple sequence alignment of cyclophilin proteins from *Triticum aestivum* (TaCypA-1), *Cajanus cajan* (CcCyp), *Camellia sinensis* (CsCyp), *Arabidopsis thaliana* (AtCyp18-3, AtCyp19-2, AtCyp19-1, AtCyp18-4, AtCyp19-3, AtCyp20-3), *Thellungiela halophila* (ThCyp1), *Oryza sativa* (OsCyp2), *Schistosoma mansoni* (SmCypA) and *Homo-sapiens* (hCypA) was performed using clustalw2 server (http://www.ebi.ac.uk/Tools/msa/clustalw2/). The ESPript 3.0 multiple-alignment editor (http://espript.ibcp.fr/ESPript/ESPript/) was used for the final presentation. The positions of the various α-helices and β-sheets in reference to the TaCypA-1 crystal structure (4HY7) are indicated at the top of the figure in red. Names of displayed cyclophilins sequences under investigation (AtCyp19-3 and TaCypA-1) have been colored red while for SmCypA and CsCyp are colored blue. Active site residues are marked by blue asterisks while the Cys residues corresponding to TaCypA-1 are marked with red triangles below the figure. The plant-specific divergent loop residues are shaded in green colour. Relative accessibility of amino acid is displayed below the alignment block (Blue: accessible, Cyan: intermediate and white: buried). The active site residues in all the sequences are very well conserved except for AtCyp19-1 for which a portion of protein sequence containing the first three active site residues and part of the divergent loop is deleted. Four Cys residues (C-40, C-69, C-122, C-168) have been conserved in all sequences except for Cys-40 in hCypA (mutated to S) and Cys 69 in AtCyp20-3 (mutated to I). TaCypA-1 has two additional Cys residues with the one corresponding to position 126 being also observed in OsCyp2 and SmCypA.

The PPIase activity of the purified AtCyp19-3 was determined by the proteolytic cleavage assay using chymotrypsin. The p-nitroanilide residue from the peptide is cleaved by chymotrypsin only from *trans*-isomer of peptide containing proline but not the *cis*-isomer of peptide substrate. The release of p-nitroanilide from test peptide leads to a concomitant absorption increase at 390 nm [46]. Relative to the uncatalysed reaction (0.015 s^-1^), the first order rate constant increased significantly (0.036 s^-1^) in the presence of 4.34 nM of recombinant AtCyp19-3 (Fig 2A), with the increase being proportional to the amount of the purified protein tested (Fig 2B). On the contrary, addition of BSA, which was used as a negative control, had no significant effect on the rate of reaction (Fig 2A), thus, implying that the observed PPIase activity was specifically due to the presence of AtCyp19-3. PPIase activity in the cell is primarily contributed by cyclophilins and FKBPs, which are inhibited specifically by CsA and FK506, respectively [5]. Incubation of AtCyp19-3 with different concentrations of CsA resulted in complete loss of activity at 120 nM, whereas FK506, even at 20 μM, had no significant effect (Fig 2C). Since no cross-inhibition is observed between cyclophilins and FKBPs by CsA or FK506 [5], these observations, therefore, imply that the purified AtCyp19-3 is a true cyclophilin. The cyclophilins from different sources show variability in sensitivity to CsA, with inhibition constants (ki) ranging from 3.9 nM to 200 nM being reported for different plant and human proteins [29, 34, 48, 54]. The ki (18.75 nM) of CsA for AtCyp19-3 (Fig 2D), observed in this study, is consistent with these reports. The purified AtCyp19-3 depicted specific activity of 17.12 x 10^2^ nmol s^-1^ mg^-1^. The catalytic efficiency of the purified AtCyp19-3 was 4.88 x 10^6^ M^-1^ s^-1^, which is comparable to that for orthologs from other plant taxa, TaCypA-1 (2.32 x 10^5^ M^-1^ s^-1^) [29], OsCyp2 (4.5 x 10^6^ M^-1^ s^-1^) [23] and CsCyp (5.6 x 10^6^ M^-1^ s^-1^) [31]. The truncated AtCyp19-3_(35–176)_ and AtCyp19-3_(71–176)_ proteins did not show significant PPIase activity (Fig 2A), which supports the *in silico* prediction (http://prosite.expasy.org/cgi-bin/prosite/mydomains/) for the presence of a PPIase domain ranging from 12 to 169 amino acid (a.a.) residues in AtCyp19-3 (Fig 3). Our observations are also consistent with the earlier studies in Arabidopsis [10] that showed that the residues arginine (R), phenylalanine (F) and histidine (H), which correspond to positions -62, -67 and -133 in AtCyp19-3, are essential for PPIase activity, and tryptophan at position 128 is required for CsA binding.

**Fig 4:**
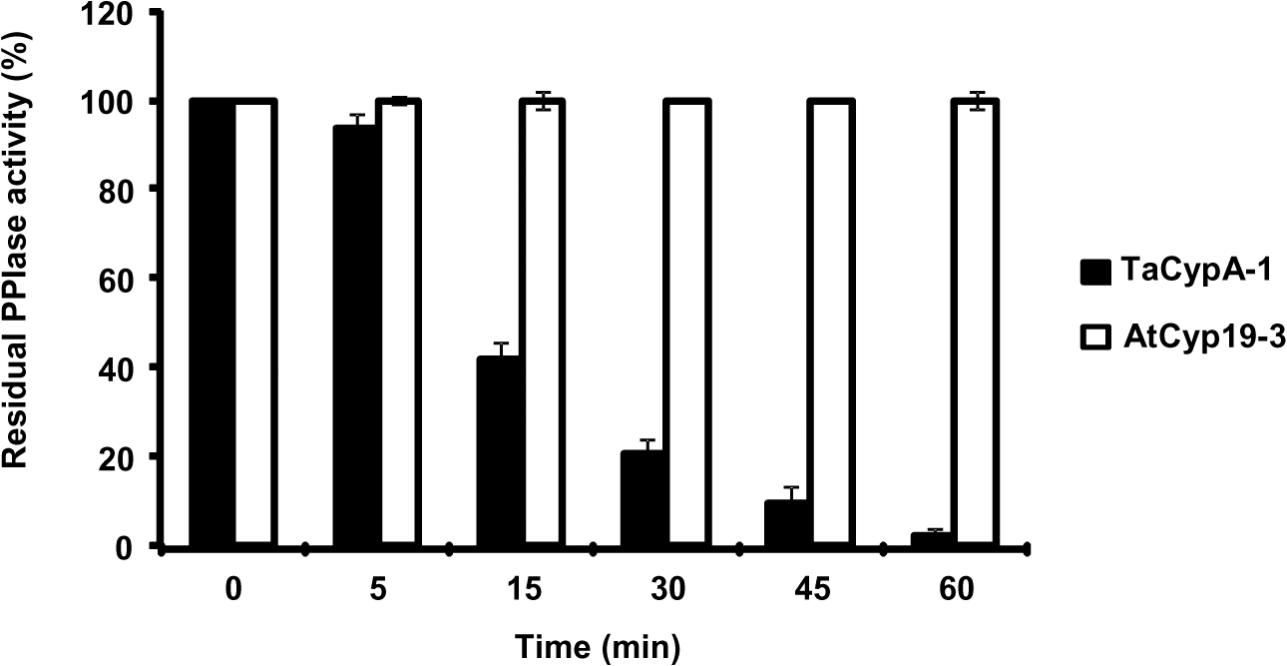
Effect of N-ethylmaleimide (500 μM) on peptidyl prolyl *cis-trans* isomerase (PPIase) activity in the presence of 22 nM each of AtCyp19-3 and TaCypA-1. The residual PPIase activity was calculated as a percentage of the initial activity before the addition of N-ethylmaleimide and expressed as a function of incubation time.

**Fig 5:**
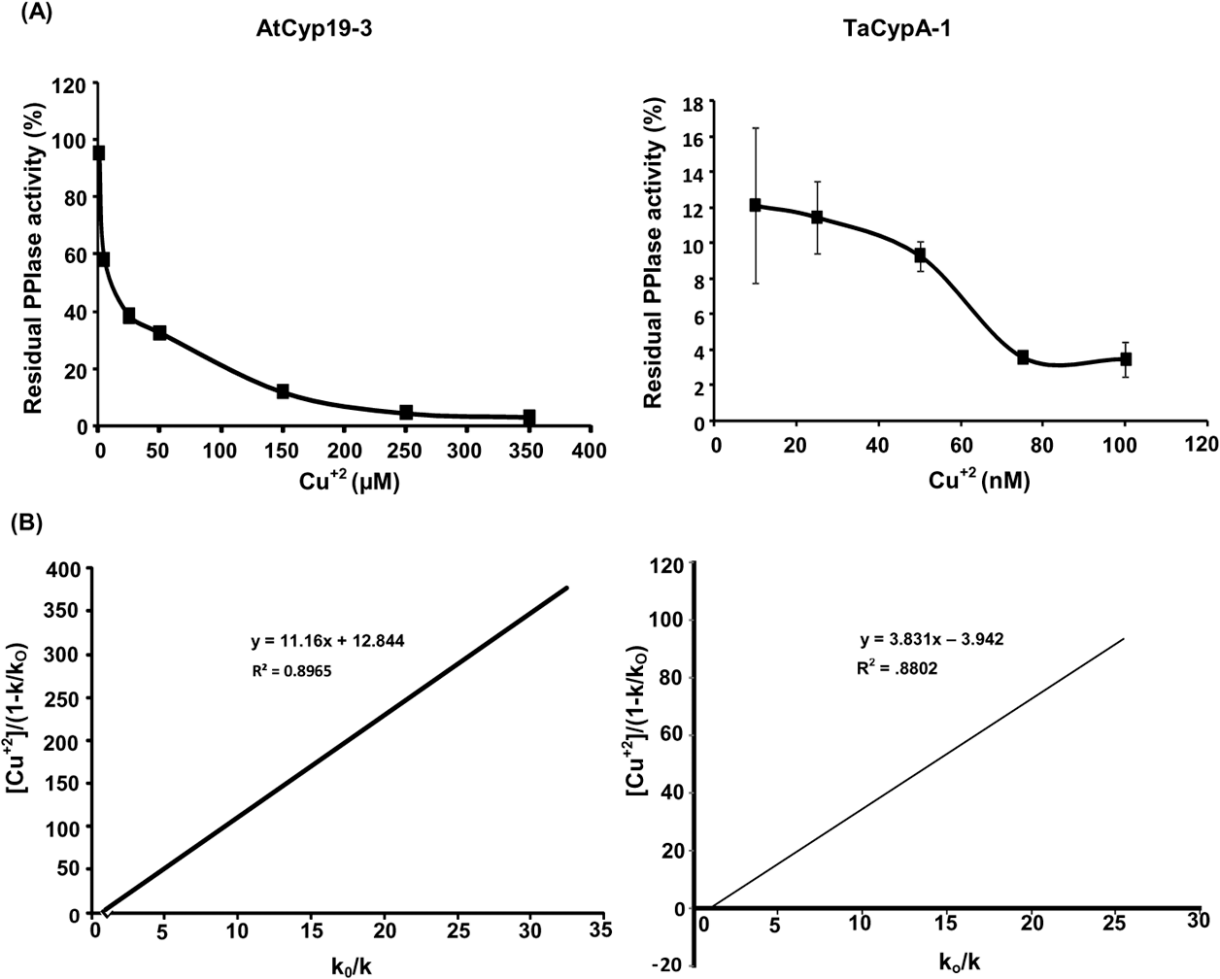
Effect of copper on the peptidyl prolyl *cis-trans* isomerase (PPIase) activity of purified recombinants AtCyp19-3 and TaCypA-1 (A) Effect of Cu^2+^ on PPIase activity of AtCyp19-3 and TaCypA-1. First order rate constants were analysed using GraFit 4.0 software. The residual PPIase activity (%) is relative to uninhibited control. **(B)** Inhibition constants (k_i_) of Cu^2+^ for AtCyp19-3 and TaCypA-1 were determined as gradient of the line of the best fit from a plot of [Cu^2+^]/(1-k/k_o_) against k_o_/k, where k is the rate constant at any given Cu^2+^ concentration and k_o_ is the rate constant in the absence of heavy metal. The slope of the line represents the ki. Data represent the mean ± SD of triplicates.

**Fig 6:**
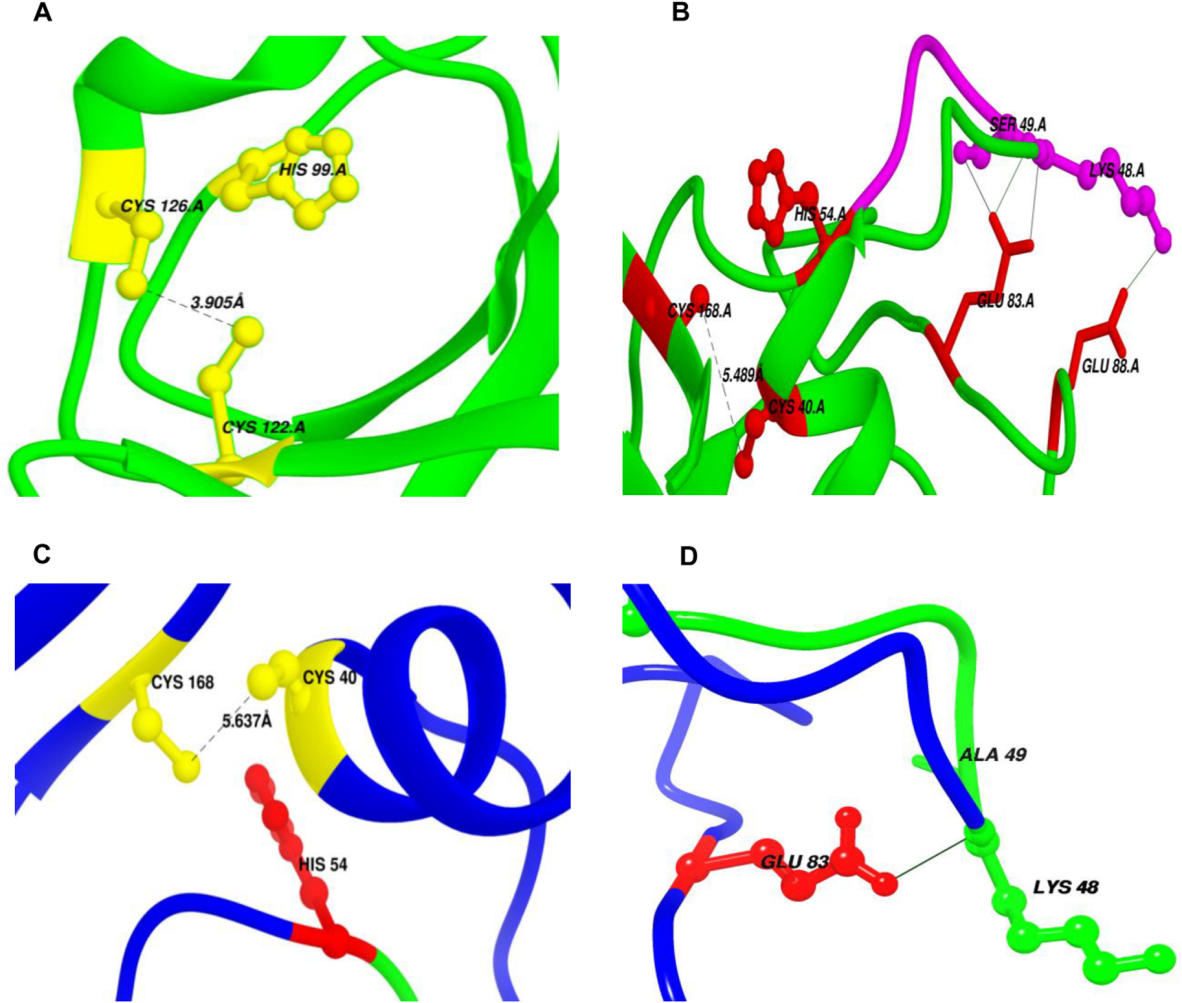
Redox control mechanisms in AtCyp19-3 and TaCypA-1 (A) Potential metal binding site (Site1) in TaCypA-1 comprising of Cys-122, His-99 and Cys-126 amino acids (yellow) as predicted by TEMSP. Cys-122-Cys-126 pair (3.905 *Å*) in TaCypA-1 which may be involved in Redox 2-Cys mechanism similar to SmCypA, is also shown. **(B)** Second potential metal binding site (Site 2) consisting of Cys-40, His-54 and Cys-168 (red). Disulfide bond between Cys-40 and Cys-168 (5.489 *Å*), and hydrogen bond network linking side chains of Glu-83(red) with divergent loop residues Lys-48 and Ser-49 (magenta) in TaCypA-1 are implicated in allosteric control similar to Redox 2-Cys Mechanism in CsCypA. **(C)** Cartoon showing His-54 and disulfide bridge forming pair Cys-40-Cys-168 (5.4 *Å*) in AtCyp19-3. This triad (yellow) may form a metal binding site in AtCyp19-3. **(D)** Hydrogen bond connecting side chain carboxyl group of Glu-83(red) with main chain amide group of Lys-48 (green) from the divergent loop in AtCyp19-3. Formation of Cys-40-Cys-168 disulfide bond disrupts this interaction and closes the active site. Distances have been shown as black dashed lines while hydrogen bonds as black solid lines.

AtCyp19-3 belongs to a sub-group of cyclophilins, termed divergent cyclophilins due to the presence of an additional loop consisting of the consensus sequence XXGKXLH (corresponding to 48–54 a.a. residues in AtCyp19-3), along with two conserved Cys-residues (Cys-40 and Cys-168) and a conserved glutamate, Glu-83. Multiple sequence alignment of a.a sequences of the divergent (TaCypA-1, CsCyp and AtCyp19-3) and non-divergent (SmCypA and AtCyp20-3) cyclophilins revealed that AtCyp19-3 shares approximately 70% identity with its wheat homologue TaCypA-1 (Fig 3) which was the first enzymatically active plant cyclophilin whose crystal structure was elucidated [29]. Except for hCypA, which has Ser instead of Cys-40, all cyclophilins (including AtCyp19-3) compared in this alignment showed three invariable Cys residues corresponding to positions 40, 122 and 168 of TaCypA-1 (Fig 3). Compared to AtCyp19-3, TaCypA-1 has additional Cys-126 and Cys-159 residues. Previous studies demonstrated that PPIase activity of several cyclophilins *viz*., CsCyp [31], SmCypA [47] and AtCyp20-3 [55, 56] was inhibited under oxidative conditions, thus, implying that the thiol groups are involved in redox regulation of these proteins. Given that AtCyp19-3 contains four Cys residues (40, 69, 122, 168) as compared to six (-40, -69, -122, -126, -159, -168) in TaCypA-1 (Fig 3), and shares 70% identity with the latter, the role of -SH groups in regulation of enzymatic activity was analyzed by determining the effect of NEM and Cu^2+^ on PPIase activity of both the proteins. NEM and Cu^2+^ both are reported to inactivate the enzymes due to interaction with-SH groups in the active site [57, 58]. The *E*.*coli* cyclophilin PpiA lacks cysteine [40] and hence, included as a negative control in this study. All the three purified cyclophilins (AtCyp19-3, TaCypA-1 and PpiA) were incubated with NEM for different durations followed by estimation of PPIase activity. No significant decrease was observed in PPIase activity of AtCyp19-3 even after 1 h of incubation with NEM (Fig 4). In fact, AtCyp19-3 activity was insensitive to NEM even after 4 h (data not shown). On the contrary, the PPIase activity of TaCypA-1 started to decline within 5 min of treatment and up to 80% of the activity was lost after 1 h (Fig 4). Incubation with Cu^2+^ resulted in significant inhibition of enzymatic activity of both AtCyp19-3 and TaCypA-1 (Fig 5A) with inhibition constants (K_i_) of 11.16 μM and 3.83 nM, respectively (Fig 5B), thus, implying that TaCypA-1 was also more sensitive to metal ions. Neither Cu^2+^ nor NEM had any significant effect on PPIase activity of PpiA (S1D and S1E Fig), thereby supporting the role of-SH groups in the regulation of AtCyp19-3 and TaCypA-1. The Cu^2+^-induced inactivation of PPIase activity was partially reversible since addition of EDTA (1 mM) and DTT (10 mM) restored about 70% and 50% activity of TaCypA-1 and AtCyp19-3, respectively (S2 Fig). The differential effect of NEM and Cu^2+^ and restoration of Cu^2+^-induced decrease in activity by EDTA and DTT is consistent with similar observations reported for phosphoenolpyruvate carboxylase [58] and rat phosphatase [59]. The relative frequency of sites utilized by the heavy metals, including Cu^2+^, is reported to be in the order: His>Cys>Asp>Glu [60]. Based on the structure of TaCypA-1 [29], models predicting the arrangement of Cys-40, Cys-168 and His-54 residues in both TaCypA-1 and AtCyp19-3 were generated (Fig 6). The arrangement of these Cys residues is invariably conserved in divergent loop cyclophilins and has been proposed as a metal binding site in *Caenorhabditis elegans* cyclophilin 3 (CyP3) [61]. *In silico* analysis using TEMSP server suggests the presence of an additional metal binding site consisting of Cys-122, Cys-126 and His-99 also in TaCypA-1 (Fig 6). Although, no evidence at the structural level has experimentally demonstrated the coordination of metal ions at these predicted binding sites, the inhibitory effect of Cu^2+^ on cyclophilin catalytic activity is well documented in CsCyp [31] and SmCypA [47]. A similar geometrical arrangement involving one histidine and two cysteines, however, has been seen in X-ray structure of the zinc complex of alcohol dehydrogenase [62]. The higher sensitivity of TaCypA-1 to Cu^2+^, therefore, may be due to the presence of two putative Cu^2+^-binding sites compared to one in AtCyp19-3. As reported earlier for SmCypA [47], the decrease in PPIase activity of AtCyp19-3 and TaCypA-1 after oxidation with Cu^2+^ may be due to the collapse of an internal pocket which contains amino acid residues critical for PPIase activity. Inhibition of PPIase activity of AtCyp19-3 and TaCypA-1 by Cu^2+^, and of TaCypA-1 by NEM also suggests that as observed for CsCyp [31] and SmCypA [47], redox regulation may play an important role in controlling the activities of AtCyp19-3 and TaCypA-1. Redox regulation of AtCyp19-3 and TaCypA-1 is further substantiated by the fact that PPIase activity of cysteine-lacking cyclophilin PpiA was not affected by Cu^2+^ or NEM (S1D and S1E Fig).

Two different models have been proposed for regulation of catalytic activity of cyclophilins by redox 2-Cys mechanism. The mechanism regulating the activity of a divergent cyclophilin, CsCyp, works in an allosteric manner [31] and entails formation of a disulfide bond between Cys-40 and Cys-168 upon oxidation. Formation of the disulfide bond between Cys-40 and Cys-168 induces a conformational change that disrupts the interaction of the divergent loop residues and catalytic loops maintained through the key Glu-83, Lys-48 and Ser-49 residues, thereby, closing the active site and consequently abrogating the PPIase activity. In AtCyp19-3, position 49 is occupied by an Ala residue rather than a Ser (Fig 3). It is likely that Lys-48 may alone be sufficient to maintain the interaction between the divergent and catalytic loops in this protein. On the contrary, the redox 2-Cys regulation of PPIase activity of a non-divergent cyclophilin, SmCypA, was attributed to the formation of disulfide bond between Cys-122 and Cys-126 upon oxidation which resulted in the loss of activity [40]. Compared to Cys-126, which is often replaced by a Thr, Cys-122 is reported to be conserved among cyclophilins across taxa [47]. Multiple sequence alignment of divergent cyclophilins revealed that TaCypA-1 contains Cys at both -122 and -126 positions, whereas AtCyp19-3 shows presence of Cys only at position -122 (Fig 3). Since presence of NEM resulted in inhibition of PPIase activity of only TaCypA-1 and not AtCyp19-3, it is likely that disulphide bond formation between Cys-122 and Cys-126 is critical for regulation of the former cyclophilin, whereas the activity of the latter may primarily be controlled through interaction between the divergent- and catalytic loops. To further understand the implications of these observations, we compared the modeled structure of AtCyp19-3 with the 3D crystal structure of TaCypA-1 (4E1Q) [29] (Fig 6). These studies revealed that the AtCyp19-3 model is similar to its template structure for CsCyp (4JJM chain A) with all the active site residues conserved, which suggests that AtCyp19-3 may function in a manner similar to that shown for CsCyp [31]. On the contrary, TaCypA-1 depicts characteristic features of both the known redox 2-Cys mechanisms, since Cys-40 and Cys-168 are identical to a corresponding disulfide bond forming pair in CsCyp, whereas, Cys-122 and Cys-126 are identical to disulfide bridge forming pair in SmCypA (Fig 6). These observations have prompted us to speculate that the activity of TaCypA-1 may be regulated through a dual mechanism involving both loop displacement as well as interaction between Cys-122 and Cys-126, thereby presenting the possibility of a unique type of allosteric regulation, not observed either in CsCyp [31] or SmCypA [47]. However, further studies involving site-directed mutagenesis and estimation of reactive thiols are needed to validate the proposed model. Our studies also suggests that the redox regulation of AtCyp19-3 may be different from the mechanism proposed for chloroplast-localized AtCyp20-3 since, as compared to cysteines at positions 40 and 168 [31] in the former, the regulation of activity of the latter has been demonstrated to be affected by disulphide bond formation between cysteines at position 63 and 186, respectively in AtCyp20-3 [55], which corresponds to positions 40 and 163, respectively in AtCyp19-3 (Fig 3). The cysteine residue corresponding to position 186 in AtCyp20-3 is lacking in all other cyclophilins analysed (Fig 3), suggesting that distinct regulatory mechanisms have evolved in these proteins, signifying the specific roles performed by different members of this family in the cell.

**Fig 7:**
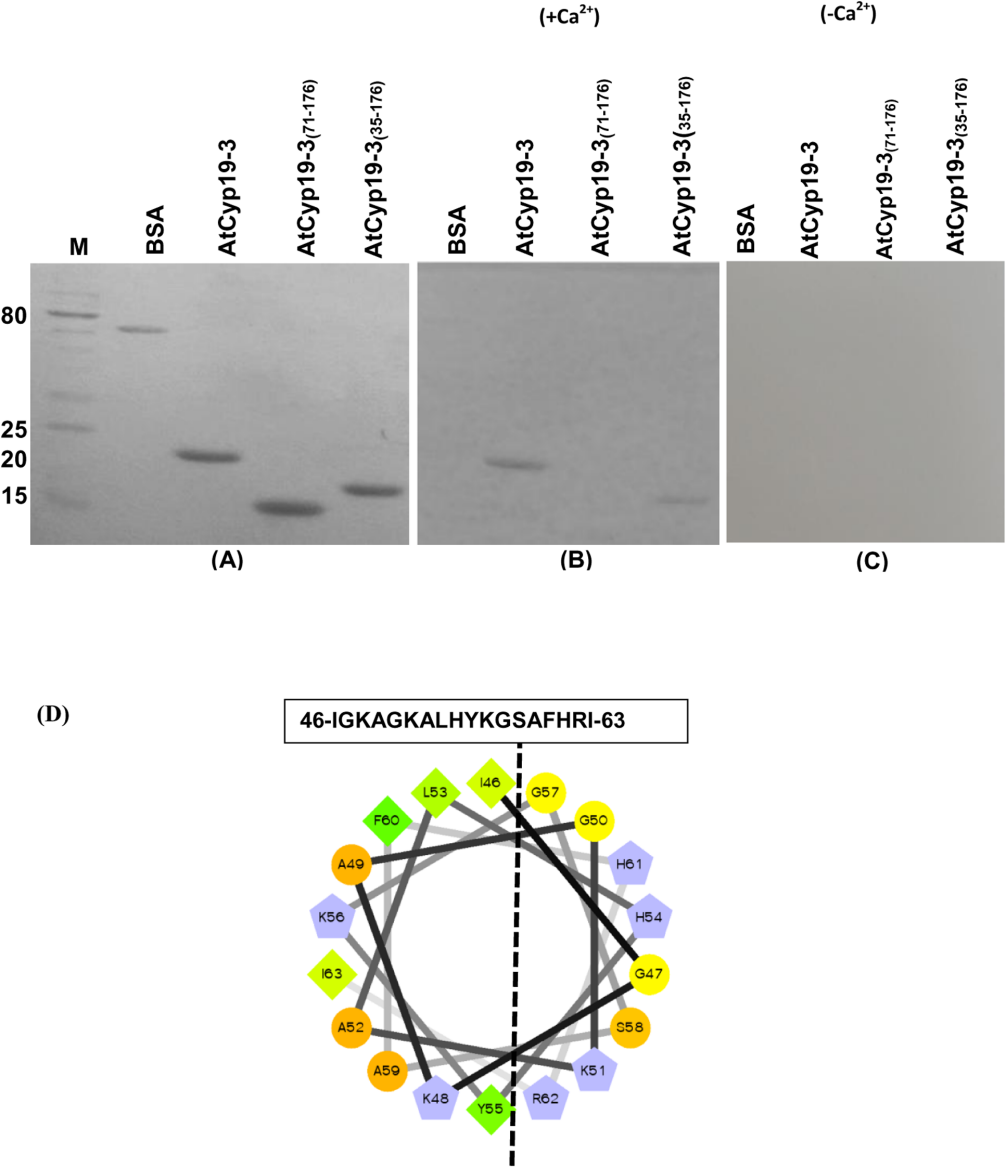
Calmodulin (CaM) gel-overlay assay of the purified recombinant AtCyp19-3 and its truncated versions. The purified proteins were resolved by using 15% SDS-PAGE followed by transfer on to Hybond C nitrocellulose membrane. Proteins transferred on to membrane, after Ponceau S staining **(A)**, were allowed to renature in the presence **(B)** and absence of Ca^2+^ **(C)** for 16 h at 4°C. The membrane was incubated with biotinylated-CaM in the presence (+Ca^2+^) and absence of Ca^2+^(-Ca^2+^), followed by probing with streptavidin-alkaline phosphate conjugate. CaM-binding proteins were visualized using nitro-blue tetrazolium/5-bromo-4-chloro-3-indolyphosphate as substrate. **(D)** Helical wheel projection (http://rzlab.ucr.edu/scripts/wheel/wheel.cgi) of the predicted CaM-binding domain (CaMBD) of AtCyp19-3. The dashed line separate the hydrophilic and the hydrophobic faces of the amphiphilic CaMBD. Hydrophilic residues: circles; hydrophobic residues: diamonds; potentially negatively charged: triangles; potentially positively charged: pentagons. Hydrophobic residues are depicted in green and the amount of green decreases proportionally to the hydrophobicity, with zero hydrophobicity coded as yellow. Hydrophilic residues are shown as red, with the amount of red being proportional to the hydrophilicity. Light blue represents the potentially charged residues. M-protein markers (kDa).

Perturbations in the intracellular levels of Ca^2+^, a secondary signal transducer, in response to different biotic and abiotic stimuli are detected by different Ca^2+^ sensors [63, 64]. CaM is one of the best-characterized Ca^2+^-sensors, which after binding to Ca^2+^ undergoes conformation change and regulates the activity of diverse range of CaM-binding proteins (CaMBPs). Interaction of CaM with its target proteins can be Ca^2+^-dependent, as has been observed for various plant proteins including kinases [39, 65], transcription activators [66] and heat shock protein 90 [49, 67], or Ca^2+^-independent, as was reported for neuromodulin and neurogranin [68]. Employing protein microarray and co-immunoprecipitation studies, Popescue et al. [38] provided evidence for the interaction of AtCyp19-3 with CaM although the dependency of this interaction on Ca^2+^ was not investigated. We used gel overlay assays to further characterize the CaM-binding property of the purified AtCyp19-3. The Ponceau S staining of the blots revealed similar amounts of the three recombinant proteins tested for interaction with CaM (Fig 7A). These studies demonstrated that the binding of CaM with recombinant AtCyp19-3 was Ca^2+^-dependent since the band corresponding to CaM-cyclophilin complex was observed only in the presence of Ca^2+^ (Fig 7B and 7C). These observations provide the first evidence that presence of Ca^2+^ is a requirement for the interaction of CaM with the AtCyp19-3.

The binding of CaM to a diverse range of proteins is facilitated through its interaction with the positively charged, amphiphilic *α-*helical domains and involves both hydrophobic and strong electrostatic interactions [69]. Helical wheel projections (http://rzlab.ucr.edu/scripts/wheel/wheel.cgi) of the predicted CaM-binding domain of AtCyp19-3 revealed the presence of a putative amphiphilic helix region (35–67 a.a. residues) in the N-terminus of this protein (Fig 7D). For identification of the domain(s) responsible for binding of this protein to CaM, truncated AtCyp19-3 lacking the first 34 and 70 a.a. residues (AtCyp19-3_(71–176)_ and AtCyp19-3_(35–176),_ respectively) were also subjected to gel overlay assays. Lanes containing the intact AtCyp19-3 and the deleted version AtCyp19-3_(35–176)_ showed the development of corresponding bands after gel overlay assay (Fig 7A and 7B). On the contrary, BSA (used as negative control) and AtCyp19-3 _(71–176)_ did not show interaction with CaM (Fig 7B). These observations imply that the CaM-binding domain in AtCyp19-3 lies between 35–70 a.a. residues in AtCyp19-3. Although a CaM-binding domain in the multidomain FKBPs was characterized earlier [70], this is the first study to delineate the putative CaM-binding site in a cyclophilin. However, fine mapping of this domain by site directed mutagenesis, which will be the subject of future studies, is required to determine the critical residues essential for interaction of AtCyp19-3 with CaM.

**Fig 8:**
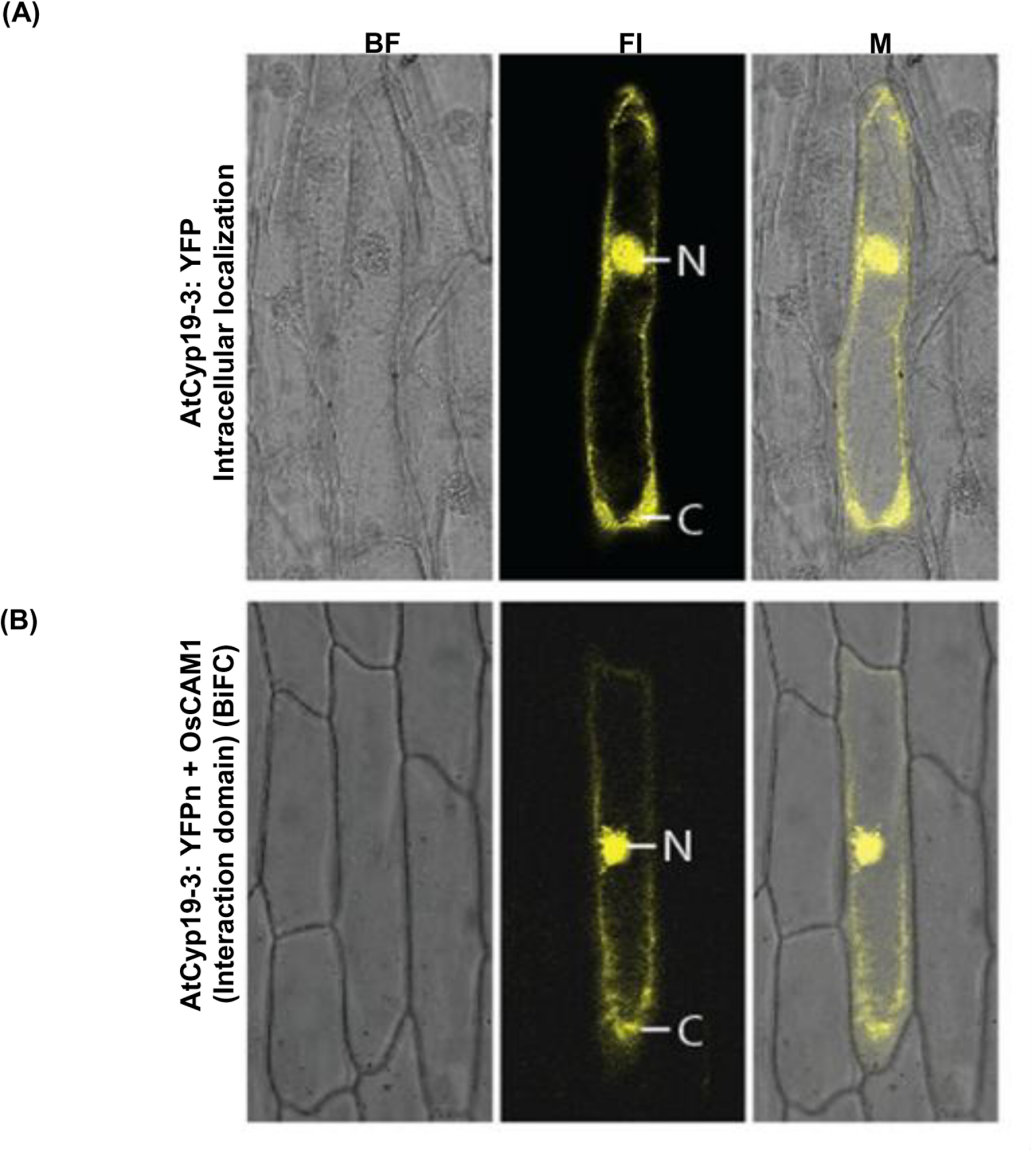
Intracellular localization of AtCyp19-3. The upper panel **(A)** shows the accumulation pattern of AtCyp19-3:YFP chimeric protein. The lower panel **(B)** shows the sub-cellular interaction of AtCyp19-3-1:YFPn and OsCaM1:YFPc chimeric proteins by bi-molecular fluorescence complementation. The chimeric polypeptide containing AtCyp19-3_(71–176),_ (which did not show binding to CaM by gel overlay assay) with YFPn served as a negative control for *in vivo* studies. BF: bright field; Fl: fluorescence image; M: merged image, N: nucleus; C:cytoplasm.

Studies on *in vivo* interaction of CaM with its target proteins in living plant cells are rare. Popescue et al. [38] provided evidence for *in vivo* interaction of AtCyp19-3 with CaM by using immunoprecipitation. However, co-immunoprecipitation requires cell lysis which may create experimental conditions that are not consistent with the natural intracellular environment and can cause fortuitous associations among proteins [71, 72]. Therefore, we employed BiFC assays to further validate the interaction of AtCyp19-3 with CaM in living cells. These observations showed that transiently expressed YFP-tagged AtCyp19-3 fusion protein was uniformly distributed in both the nucleus and the cytosol (Fig 8A). These findings are in agreement with earlier studies that reported dual localization of cyclophilins from *Thellungiella halophila* (ThCyp1) [73], *Cajanus cajan* (CcCyp) [24], *Oryza sativa* (OsCyp2) [19], which show 74%, 60% and 71% identity, respectively, with AtCyp19-3 (S2 Table). Previously, by transfection of GFP-fused proteins in *Arabidopsis* protoplasts, the rice CaM, OsCaM1, was found to localize to both cytosolic and nuclear compartments [74]. Given that AtCyp19-3 showed *in vitro* binding to CaM, and is localized to both the nucleus and cytosol, we examined the physical interaction of this protein with OsCaM, which is 99.33% identical to AtCaM7 (Q0JNS6.2) (S3 Fig), in onion peel cells transformed with AtCyp19-3:YFPn and OsCaM1:YFPc constructs. The chimeric polypeptide containing AtCyp19-3_(71–176),_ (which did not show binding to CaM by gel overlay assay) with YFPn served as a negative control for *in vivo* studies. The results of this experiment showed that AtCyp19-3 and OsCaM1 are capable of associating *in vivo* in both cytosolic and nuclear compartments, whereas AtCyp19-3_(71–176)_ did not show any interaction with CaM. These observations further validate that the CaM-binding domain in AtCyp19-3 is located in the 1–70 amino acid N-terminus region. These studies also suggest that the interaction of these proteins does not influence their respective subcellular distributions.

Since CaM is reported to regulate enzymatic activity of its target proteins after binding [65], therefore, changes in PPIase activity of AtCyp19-3 were studied in the presence of 5- and 10-fold molar excess of CaM. The presence of CaM had no significant effect on PPIase activity of the purified cyclophilin, suggesting that CaM may not be involved in direct regulation of enzymatic activity of this protein (S4 Fig). Besides *cis* to *trans* isomerization, the plant cyclophilins are also implicated in other cellular functions through their interaction with other proteins [18–20]. It is, therefore, likely that as observed for a rice CaM-binding protein kinase, OsCBK, CaM may be regulating functions of AtCyp19-3, which are independent of enzymatic activity. The role of CaM in regulation of AtCyp19-3 is also supported by the fact that the two proteins show co-localization but further studies are required to elucidate the precise physiological implications of AtCyp19-3 and CaM interaction.

Plant cyclophilins participate in several cellular functions through their interaction with other proteins [19–20]. Expression of cyclophilins is reported to enhance under different biotic and abiotic stress conditions and these proteins have been implicated in stress adaptation response of plants [21–24, 75]. Since modulation of proteins by Ca^2+^-CaM is one of the important regulatory mechanisms by which the cells respond to external cues [64, 76], it is likely that activity(ies) of AtCyp19-3 may be controlled through Ca^2+^/CaM pathway, which however needs to be investigated further.

To conclude, the present study is the first to demonstrate that AtCyp19-3 is an enzymatically active cyclophilin and its PPIase activity appears to be regulated by a redox-based mechanism. Further, evidence is provided for the interaction of AtCyp19-3 with CaM *in vitro* and *in vivo* using gel-overlay and BiFC assays, respectively. These studies revealed that interaction of AtCyp19-3 with CaM is Ca^2+^-dependent, and the putative CaM-binding domain comprises of 35–70 amino acid residues in the N-terminus region of this protein. However, PPIase activity of AtCyp19-3 does not appear to be affected by CaM *in vitro*, suggesting a novel CaM-mediated role for this cyclophilin family member in Ca^2+^ signalling.

## Supporting Information

S1 Fig (A) SDS-PAGE analysis of purified recombinant PpiA. Total protein was isolated from recombinant *E*.*coli* BL21(DE3) RIL before (UI) and after induction (I) with 0.5 mM IPTG, and purified by Ni-NTA affinity column. Arrow indicates the purified recombinant protein. (B) Purified protein transferred on to Hybond C membrane followed by Ponceau S staining. (C) Confirmation of the purified recombinant protein (arrow) by immunoblotting with the anti-His antibodies. M: markers. (D) Hydrolysis of N-succinyl-ala-ala-pro-phe-p-nitroanilidine (peptidyl prolyl *cis-trans* isomerase or PPIase activity) in the presence of 52.2 nM recombinant PpiA. The rate of reaction is expressed as first order rate constant k (s^-1^). (E) Effect of Cu^2+^ and NEM on the PPIase activity of purified *E*.*coli* PpiA (Cyp). The purified PpiA (52.2 nM) was incubated with 250 μM Cu^2+^, 500 μM CsA [41] and 500 μM NEM before carrying out PPIase assays. Data represent the mean ± S.D of three replicates.
(TIF)

S2 Fig Effect of EDTA (1mM) and DTT (10 mM) on the Cu^2+^-induced inhibition of peptidyl prolyl *cis-trans* isomerase (PPIase) activity of TaCypA-1 and AtCyp19-3. The decrease in PPIase activity was calculated relative to uninhibited control.
(TIF)

S3 Fig Alignment of calmodulin proteins. Multiple sequence alignment of calmodulins of *Arabidopsis thaliana* (AtCaM7) and *Oryza sativa* (OsCaM1) was performed using clustalw2 server(http://www.ebi.ac.uk/Tools/msa/clustalw2/).
(TIF)

S4 Fig Effect of calmodulin (CaM) on peptidyl prolyl *cis-trans* isomerase (PPIase) activity of AtCyp19-3 (Cyp). The purified AtCyp19-3 (22 nM) was incubated with CaM (100 nM and 200 nM) at 25°C for 4 h before carrying out PPIase assays. Bovine serum albumin (BSA) (200 nM) was used as a control. Data represent the mean ± S.D of three replicates.
(TIF)

S1 Table Primers used for amplification of the full length *AtCyp19-3* and its truncated versions and *E*.*coli ppiA*. The restriction sites are underlined.
(PDF)

S2 Table Identity and similarity analysis. Percentage identity and similarity generated by MATGAT V 2.0 software for plant cyclophilins from different organism viz. *Triticum aestivum* (TaCypA-1), *Cajanus cajan* (CcCyp), *Camellia sinensis* (CsCyp), *Arabidopsis thaliana* (AtCyp18-3, AtCyp19-2, AtCyp19-1, AtCyp18-4, AtCyp19-3, AtCyp20-3), *Thellungiela halophila* (ThCyp1), *Oryza sativa* (OsCyp2) and *Schistosoma mansoni* (SmCypA).
(PDF)
