# Supplementary figures and images for: Characterization of Peptidyl-Prolyl Cis-Trans Isomerase- and Calmodulin-Binding Activity of a Cytosolic Arabidopsis thaliana Cyclophilin AtCyp19-3
Source: PLoS One. 2015 Aug 28;10(8):e0136692. doi: 10.1371/journal.pone.0136692 (PMC4552658; doi:10.1371/journal.pone.0136692)

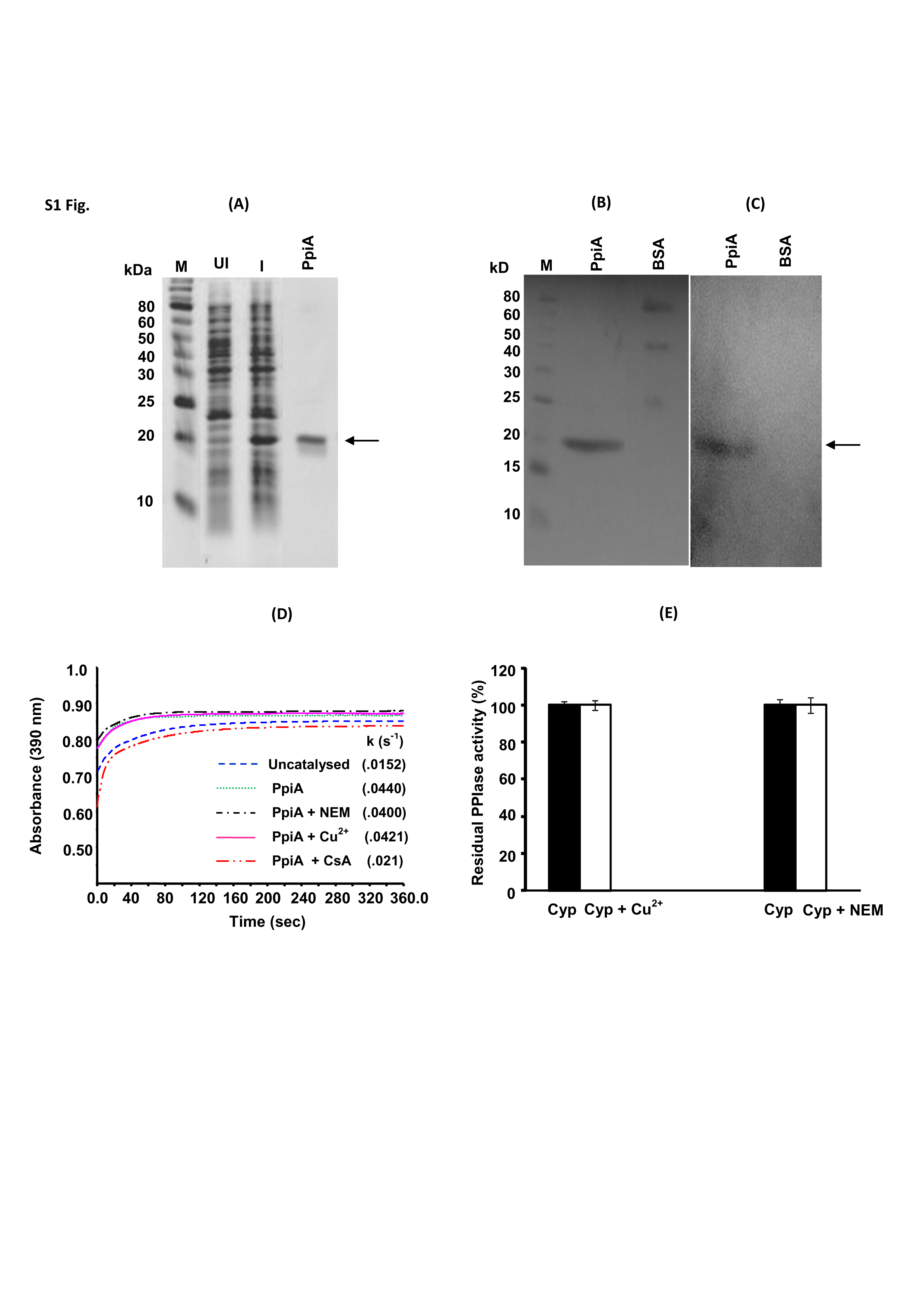

Supplement: S1 Fig — Total protein was isolated from recombinant E.coli BL21(DE3) RIL before (UI) and after induction (I) with 0.5 mM IPTG, and purified by Ni-NTA affinity column. Arrow indicates the purified recombinant protein. (B) Purified protein transferred on to Hybond C membrane followed by Ponceau S staining. (C) Confirmation of the purified recombinant protein (arrow) by immunoblotting with the anti-His antibodies. M: markers. (D) Hydrolysis of N-succinyl-ala-ala-pro-phe-p-nitroanilidine (peptidyl prolyl cis-trans isomerase or PPIase activity) in the presence of 52.2 nM recombinant PpiA. The rate of reaction is expressed as first order rate constant k (s-1). (E) Effect of Cu2+ and NEM on the PPIase activity of purified E.coli PpiA (Cyp). The purified PpiA (52.2 nM) was incubated with 250 μM Cu2+, 500 μM CsA [41] and 500 μM NEM before carrying out PPIase assays. Data represent the mean ± S.D of three replicates. (TIF) [file pone.0136692.s001.tif]

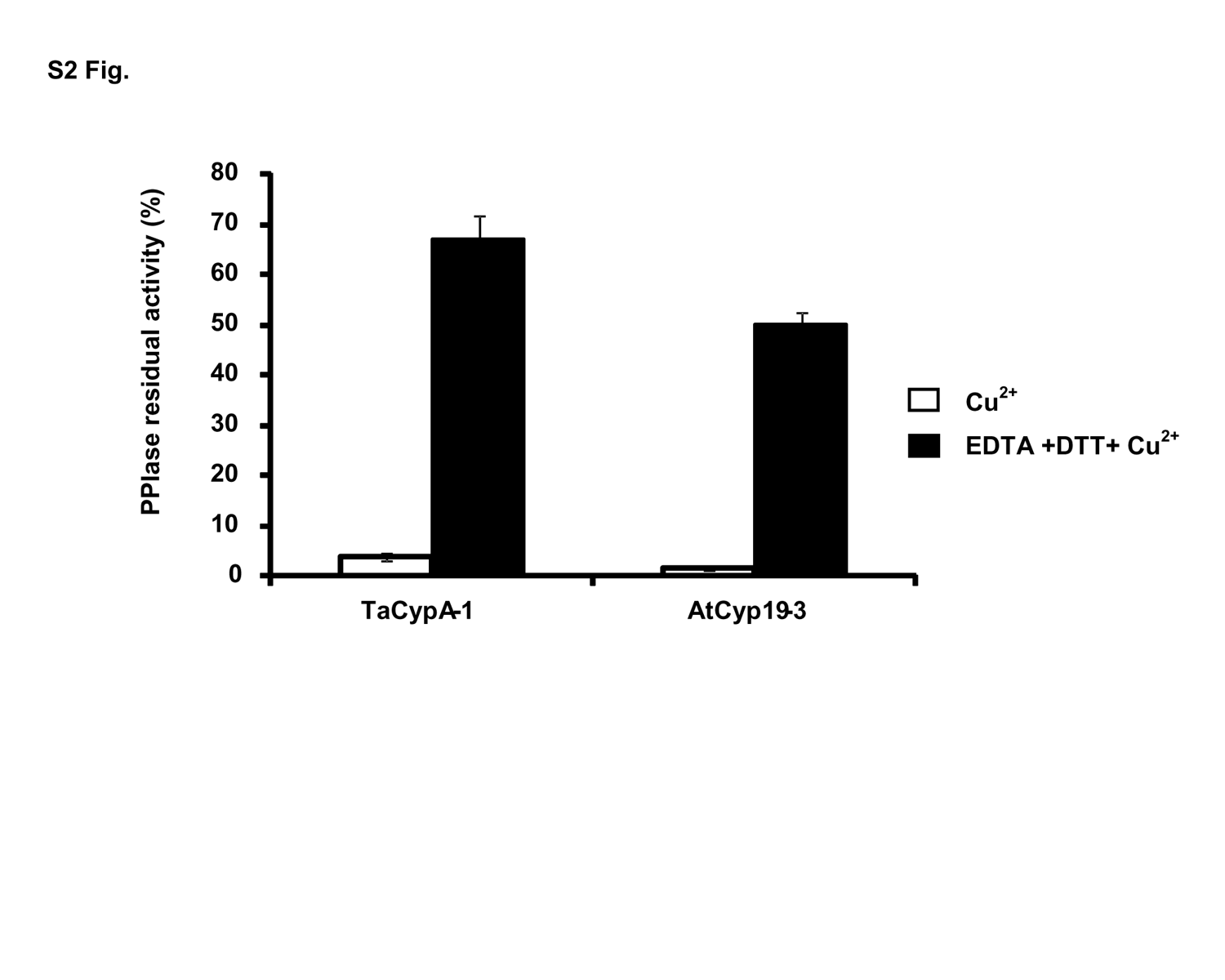

Supplement: S2 Fig — The decrease in PPIase activity was calculated relative to uninhibited control. (TIF) [file pone.0136692.s002.tif]

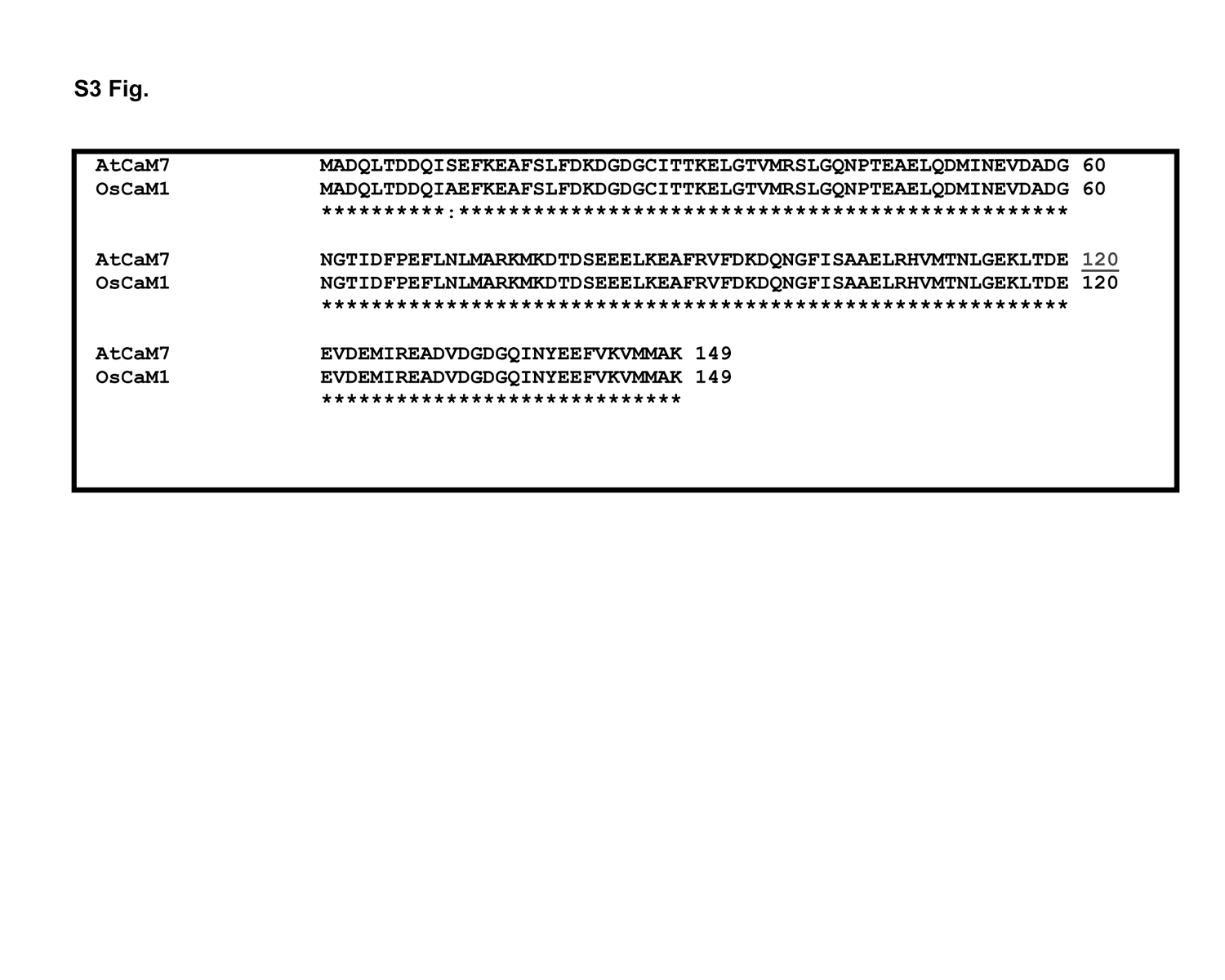

Supplement: S3 Fig — Multiple sequence alignment of calmodulins of Arabidopsis thaliana (AtCaM7) and Oryza sativa (OsCaM1) was performed using clustalw2 server(http://www.ebi.ac.uk/Tools/msa/clustalw2/). (TIF) [file pone.0136692.s003.tif]

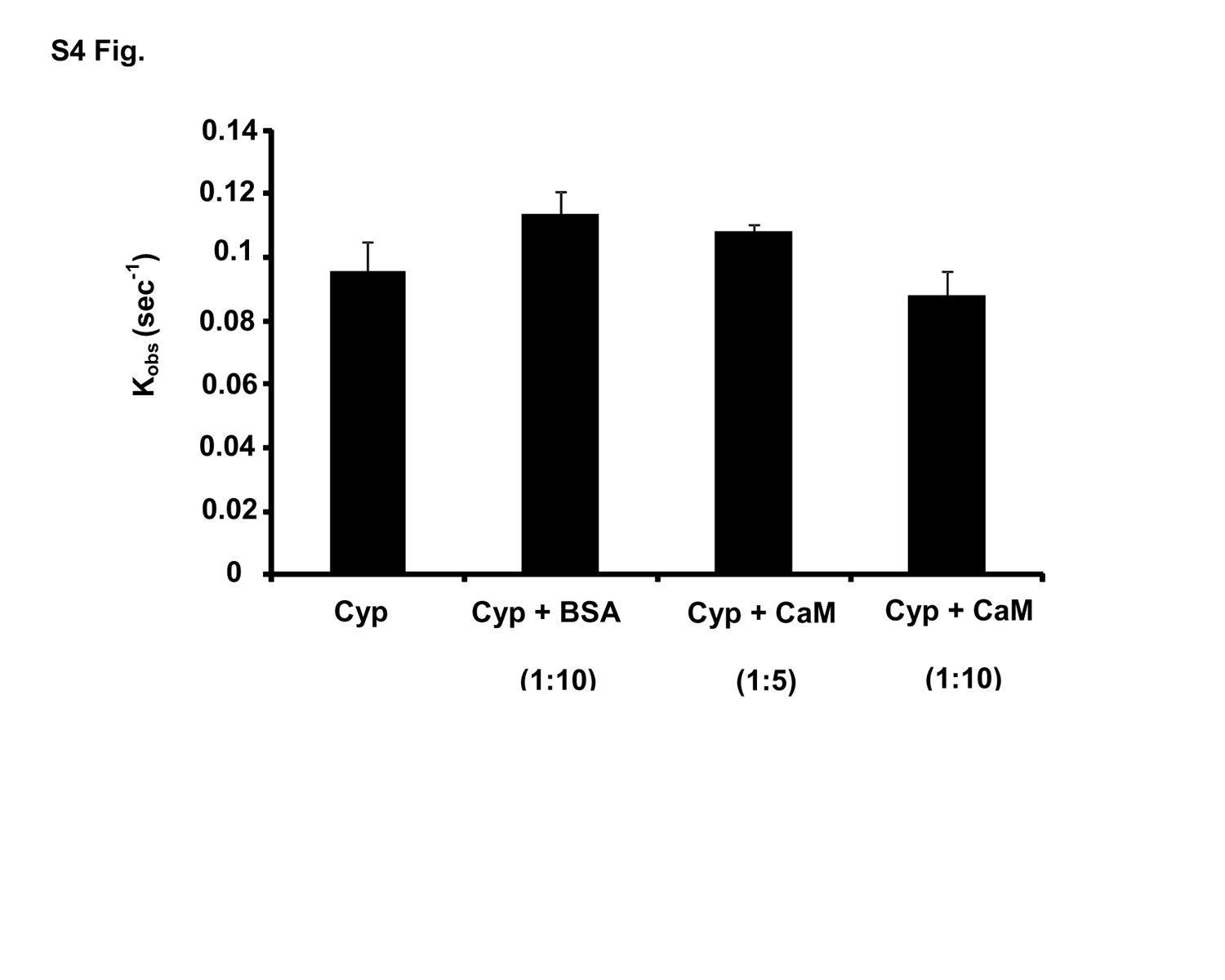

Supplement: S4 Fig — The purified AtCyp19-3 (22 nM) was incubated with CaM (100 nM and 200 nM) at 25°C for 4 h before carrying out PPIase assays. Bovine serum albumin (BSA) (200 nM) was used as a control. Data represent the mean ± S.D of three replicates. (TIF) [file pone.0136692.s004.tif]
