# Supplementary material for: Characterization of Peptidyl-Prolyl Cis-Trans Isomerase- and Calmodulin-Binding Activity of a Cytosolic Arabidopsis thaliana Cyclophilin AtCyp19-3
Source: PLoS One. 2015 Aug 28;10(8):e0136692. doi: 10.1371/journal.pone.0136692 (PMC4552658; doi:10.1371/journal.pone.0136692)
Supplement: S1 Table — The restriction sites are underlined. (PDF) [file pone.0136692.s005.pdf]

S1 Table: Primers used for amplification of the full length *AtCyp19-3* and its truncated versions and *E.coli ppiA*. The restriction sites are underlined.

Protein	Primer	Annealing temperature (˚C)	Amplicon (bp)
AtCyp19-3	F: CG<u>GAATTC</u>ATGGCGAATCCTAAAGTCTTC<br>R:CCG<u>CTCGAG</u>TTATGAACTTGGGTTCTTGAG	46	528
AtCyp $_{(71\text{-}176)}$	F: CGG<u>GAATTC</u>GGT GGAGATTTCACTCGTG<br>R: CCG<u>CTCGAG</u>TTATGAACTTGGGTTCTTGAG	51	315
AtCyp $_{(35\text{-}176)}$	F:CGG<u>GAATTC</u>AATTTCCGTGCTTTGTGCACT<br>R:CCG<u>CTCGAG</u>TTATGAACTTGGGTTCTTGAG	52	423
PpiA	F: AAT<u>GGATCC</u>ATGTTCAAATCGACCCTGGC<br>R: TAA<u>CTCGAG</u>CGGCAGGACTTTAGCGG	62	570
