# Supplementary material for: Characterization of Peptidyl-Prolyl Cis-Trans Isomerase- and Calmodulin-Binding Activity of a Cytosolic Arabidopsis thaliana Cyclophilin AtCyp19-3
Source: PLoS One. 2015 Aug 28;10(8):e0136692. doi: 10.1371/journal.pone.0136692 (PMC4552658; doi:10.1371/journal.pone.0136692)
Supplement: S2 Table — Percentage identity and similarity generated by MATGAT V 2.0 software for plant cyclophilins from different organism viz. Triticum aestivum (TaCypA-1), Cajanus cajan (CcCyp), Camellia sinensis (CsCyp), Arabidopsis thaliana (AtCyp18-3, AtCyp19-2, AtCyp19-1, AtCyp18-4, AtCyp19-3, AtCyp20-3), Thellungiela halophila (ThCyp1), Oryza sativa (OsCyp2) and Schistosoma mansoni (SmCypA). (PDF) [file pone.0136692.s006.pdf]

S2 Table: Percentage identity and similarity generated by MATGAT V 2.0 software for plant cyclophilins from different organism viz. *Triticum aestivum* (TaCypA-1), *Cajanus cajan* (CcCyp), *Camellia sinensis* (CsCyp), *Arabidopsis thaliana* (AtCyp18-3, AtCyp19-2, AtCyp19-1, AtCyp18-4, AtCyp19-3, AtCyp20-3), *Thellungiela halophila* (ThCyp1), *Oryza sativa* (OsCyp2) and *Schistosoma mansoni* (SmCypA).

Identity %

	**AtCyp19-3**	**TaCypA-1**	**AtCyp18-3**	**AtCyp19-1**	**AtCyp20-3**	**AtCyp18-4**	**AtCyp19-2**	**CcCyp**	**ThCyp1**	**OsCyp2**	**SmCypA**	**CsCYP**
**AtCyp19-3**		**69.9**	**72.7**	**65.0**	**41.8**	**72.7**	**72.3**	**60.2**	**74.0**	**71.0**	**62.8**	**76.1**
**TaCypA-1**	**80.7**		**79.1**	**66.5**	**41.7**	**75.0**	**79.3**	**64.0**	**80.9**	**86.6**	**61.5**	**83.1**
**AtCyp18-3**	**82.4**	**85.5**		**71.7**	**42.1**	**78.5**	**90.2**	**69.2**	**83.2**	**78.5**	**60.9**	**84.3**
**AtCyp19-1**	**73.3**	**74.3**	**79.7**		**37.8**	**69.4**	**69.5**	**56.6**	**78.0**	**66.5**	**56.4**	**73.4**
**AtCyp20-3**	**50.6**	**50.6**	**50.6**	**45.9**		**38.7**	**43.6**	**33.8**	**42.5**	**39.8**	**39.0**	**41.7**
**AtCyp18-4**	**81.8**	**82.0**	**86.6**	**79.1**	**47.9**		**77.0**	**64.5**	**81.5**	**76.2**	**59.8**	**81.4**
**AtCyp19-2**	**86.4**	**87.9**	**95.4**	**79.9**	**51.7**	**86.2**		**67.2**	**83.3**	**78.2**	**61.1**	**81.6**
**CcCyp**	**70.5**	**73.8**	**77.3**	**66.9**	**44.0**	**76.7**	**77.0**		**65.9**	**62.2**	**50.8**	**72.1**
**ThCyp1**	**85.8**	**86.7**	**90.2**	**82.7**	**51.0**	**90.2**	**94.3**	**76.3**		**80.9**	**59.8**	**85.5**
**OsCyp2**	**81.8**	**90.7**	**84.3**	**73.3**	**49.4**	**82.0**	**86.8**	**70.3**	**87.9**		**60.9**	**83.1**
**SmCypA**	**73.3**	**73.8**	**76.7**	**68.6**	**50.2**	**70.9**	**77.0**	**64.5**	**75.1**	**76.2**		**60.9**
**CsCYP**	**84.7**	**86.6**	**91.3**	**80.2**	**49.8**	**89.0**	**91.4**	**81.4**	**91.3**	**86.6**	**76.2**	

Similarity %
